# Phosphopantetheinyl transferase (Ppt)-mediated biosynthesis of lysine, but not siderophores or DHN melanin, is required for virulence of *Zymoseptoria tritici* on wheat

**DOI:** 10.1038/s41598-018-35223-8

**Published:** 2018-11-20

**Authors:** Mark C. Derbyshire, Amir Mirzadi Gohari, Rahim Mehrabi, Sreedhar Kilaru, Gero Steinberg, Solaf Ali, Andy Bailey, Kim Hammond-Kosack, Gert H. J. Kema, Jason J. Rudd

**Affiliations:** 1 BioIntercations and Crop Protection, Rothamsted Research, Harpenden, Hertfordshire, UK; 2 Centre for Crop and Disease Management, Curtin University, Perth, Australia; 3 Department of Plant Pathology, Faculty of Agricultural Sciences and Engineering, College of Agriculture and Natural Resources, University of Tehran, Karaj, Iran; 4 Wageningen University and Research, Wageningen Plant Research, PO Box 16, 6700AA Wageningen, The Netherlands; 5 Department of Biotechnology, College of Agriculture, Isfahan University of Technology, Isfahan, 84156-83111 Iran; 6 Biosciences, University of Exeter, Exeter, EX4 4QD UK; 7 School of Biological Sciences, Bristol University, 24 Tyndall Avenue, Bristol, UK; 8 Wageningen University and Research, Laboratory of Phytopathology, PO box 16, 6700AA Wageningen, The Netherlands; 9 Technical College of Health, Sulaimani Polytechnic University, Qrga, Wrme Street, Mardin 327, Alley 76, Sulaimaniyah, Kurdistan Region of Iraq, Sulaimani Governorate, Iraq

**Keywords:** Fungi, Molecular biology

## Abstract

*Zymoseptoria tritici* is the causal agent of Septoria tritici blotch (STB) disease of wheat. *Z. tritici* is an apoplastic fungal pathogen, which does not penetrate plant cells at any stage of infection, and has a long initial period of symptomless leaf colonisation. During this phase it is unclear to what extent the fungus can access host plant nutrients or communicate with plant cells. Several important primary and secondary metabolite pathways in fungi are regulated by the post-translational activator phosphopantetheinyl transferase (Ppt) which provides an essential co-factor for lysine biosynthesis and the activities of non-ribosomal peptide synthases (NRPS) and polyketide synthases (PKS). To investigate the relative importance of lysine biosynthesis, NRPS-based siderophore production and PKS-based DHN melanin biosynthesis, we generated deletion mutants of *ZtPpt*. The ∆*ZtPpt* strains were auxotrophic for lysine and iron, non-melanised and non-pathogenic on wheat. Deletion of the three target genes likely affected by ZtPpt loss of function (*Aar*- lysine; *Nrps1*-siderophore and *Pks1*- melanin), highlighted that lysine auxotrophy was the main contributing factor for loss of virulence, with no reduction caused by loss of siderophore production or melanisation. This reveals Ppt, and the lysine biosynthesis pathway, as potential targets for fungicides effective against *Z. tritici*.

## Introduction

The Ascomycete fungus *Zymoseptoria tritici* (Desm.) (Quaedvlieg & Crous)^1^ causes Septoria tritici blotch (STB) disease of wheat in temperate growing regions worldwide^2,3^. This disease is widely considered, both in industry and academia, to be the most important wheat disease in Europe^3^. It is estimated that approximately 70% of the $1.7 billion a year European fungicide market for wheat is sold for the control of STB^4^. Extensive use of fungicides has led to numerous cases of resistance in *Z. tritici*, and new chemistries are needed for future disease control^5,6^. Knowledge of essential-for-life and important virulence genes in this fungus may aid the development of these new chemistries^7^.

*Z. tritici* is often considered a hemibiotrophic fungus, as it undergoes two distinct phases of plant colonisation – an initial symptomless infection lasting at least one week, which is subsequently followed by a necrotrophic phase associated with the formation of leaf lesions. During the symptomless phase, *Z. tritici* typically enters leaves through stomata and colonizes mesophyll tissue where it grows in the intercellular space, the “apoplast”, without producing haustoria or any other form of plant cell penetrating structures. The onset of leaf necrosis is then rapid and begins with the appearance of chlorotic lesions. These eventually coalesce into necrotic blotches bearing pycnidia, which are asexual fructifications containing rain splash-dispersed pycnidiospores^8^. This entire infection cycle occurs without fungal penetration of host cells. For this reason, *Z. tritici* is also referred to as an ‘apoplastic’ pathogen, which delineates it from many other plant pathogenic fungi that do physically penetrate plant cells at some point during infection. However, the full repertoire of mechanisms that enable initial symptomless colonization and the subsequent activation of leaf necrosis by *Z. tritici* have not been fully elucidated.

Several plant pathogenic fungi produce diffusible secondary metabolites (SMs) to induce host tissue necrosis, which supports colonisation. Many of these SMs are produced by non-ribosomal peptide synthase (NRPS) or polyketide synthase (PKS) enzymes frequently residing in genome clusters with other tailoring genes and transporters. Important examples include the victorin toxin of *Cochliobolus victoriae* and the T-toxin of *C. heterostrophus*. Victorin is a non-ribosomal peptide (NRP) that is produced by the NRPS enzyme TOX3^9^. This toxin causes programmed cell death (PCD) in *Arabidopsis thaliana* by interacting with the nucleotide binding site leucine rich repeat (NB-LRR) receptor protein, LOV1^10^. T-toxin is a polyketide (PK) produced by PKS enzymes encoded at two distinct loci, Tox1A and Tox1B^11^. It interacts with mitochondria in Texas male sterile cytoplasm corn to cause necrosis^12^. Both T-toxin and victorin are essential for virulence in their respective pathogens^13^.

As bioactive molecules, SMs are often involved in a variety of other processes such as microbial competition, and melanin and siderophores are two key examples of metabolites frequently found to be produced by most fungi^14–16^. In the citrus pathogen *Alternaria alternata*, disruption of the NRPS siderophore synthetase-encoding gene *NPS6* leads to hypersensitivity to low iron availability and reactive oxygen species (ROS), which leads to reduced virulence^14^. This reduction in virulence is likely due to the iron cofactor requirement of enzymes that quench host derived ROS. In *Z. tritici* several studies have predicted the role of a specific PKS (Pks1) in melanin biosynthesis but this gene has, to date, not been functionally characterised^17,18^.

Despite diverse biological roles, all NRPSs and PKSs share a single point of posttranslational activation. To function, they must be 4′-phosphopantetheinylated at conserved serine residues within the acyl carrier protein (ACP) domain. The enzyme that carries out this process is 4′-phosphopantetheinyl transferase (PPT), which was first described for filamentous fungi in *Aspergillus nidulans*^19,20^. In addition to its role in the biosynthesis of SMs and siderophores, Ppt is essential for 4′-phosphopantetheinylation of alpha aminoadipate reductase (Aar), an enzyme crucial for lysine biosynthesis. The *Saccharomyces cerevisiae* Ppt homologue was first described as the enzyme LYS5, for which cognate mutants were auxotrophic for lysine^21^.

Following its discovery in model fungi, Ppt homologues were characterised in several plant pathogens with the aim of identifying links to pathogenicity via production of SMs. These include *Colletotrichum graminicola, Magnaporthe oryzae, Fusarium fujikuroi* and *Cochliobolus sativus*^22–24^. In all these species, Ppt mutant strains were shown to be auxotrophic for lysine and unable to melanise or produce siderophores. In addition, a Ppt homologue with similar functions has also been investigated in the endophytic fungus *Trichoderma virens*^25^. The auxotrophy for lysine of Ppt mutants has led to its consideration as a potential antifungal target. For example, in the human pathogens *Candida albicans* and *Aspergillus fumigatus*, high throughput screens have been developed with the aim of identifying inhibitors of this enzyme^26,27^.

Though some components of the interaction between *Z. tritici* and *Triticum aestivum* have been elucidated^28–30^, the biological role of SMs in *Z. tritici* and key genes implicated in the regulation of SMs have not been functionally characterised. To gain an insight into the functions of *Z. tritici* SMs and to ascertain the importance of lysine biosynthesis, siderophore biosynthesis and melanisation for the apoplastic lifestyle and virulence, the *Z. tritici* Ppt homologue *ZtPpt* was functionaly characterised by gene deletion. In addition, we generated and characterised deletion mutants for key downstream targets of Ppt that are directly responsible for melanisation (*Pks1*), siderophore (*Nrps1*) and lysine (*Aar*) biosynthesis. Further gene deletion strains were generated and characterised for the recently described *Z. tritici* transcriptional regulator StuA^31^, and two additional Polyketide synthases (PKS7 and PKS8) which previous studies had shown to be highly expressed during leaf infection^32,33^.

## Results

### Identification of *Zymoseptoria tritici* homologues of Ppt and genes implicated in regulating biosynthesis of lysine, siderophore and DHN-melanin

A BLASTp analysis was conducted to determine whether *Z. tritici* contains clear homologues of Ppt and associated genes previously characterised in *C. sativus*^22^. This showed that *Z. tritici* possesses a single Ppt homologue, a single Pks1 homologue, a single Aar homologue and two homologues for NPS6 (Supplementary Table 1). We performed additional Blastp analyses using characterised sequences from the *A*. *fumigatus* siderophore biosynthesis pathway to more accurately ascribe the best predicted functional homologue of the *Aspergillus fumigatus* protein NPS6, which revealed the protein ZtNrps1 (Supplementary Table 1). The protein encoded by *ZtNrps1* was homologous to both the *sidC* and *sidD* protein families but not to other siderophore biosynthetic proteins that are not generated by NRPSs. ZtNrps1 was in fact most similar to sidD, which is involved in the biosynthesis of an extracellular fusarin C siderophore (Supplementary Table 2).

### *Z. tritici* Ppt deletion mutants are lysine auxotrophs, hypersensitive to iron depletion and ROS, and are deficient in melanisation

**Figure 1:**
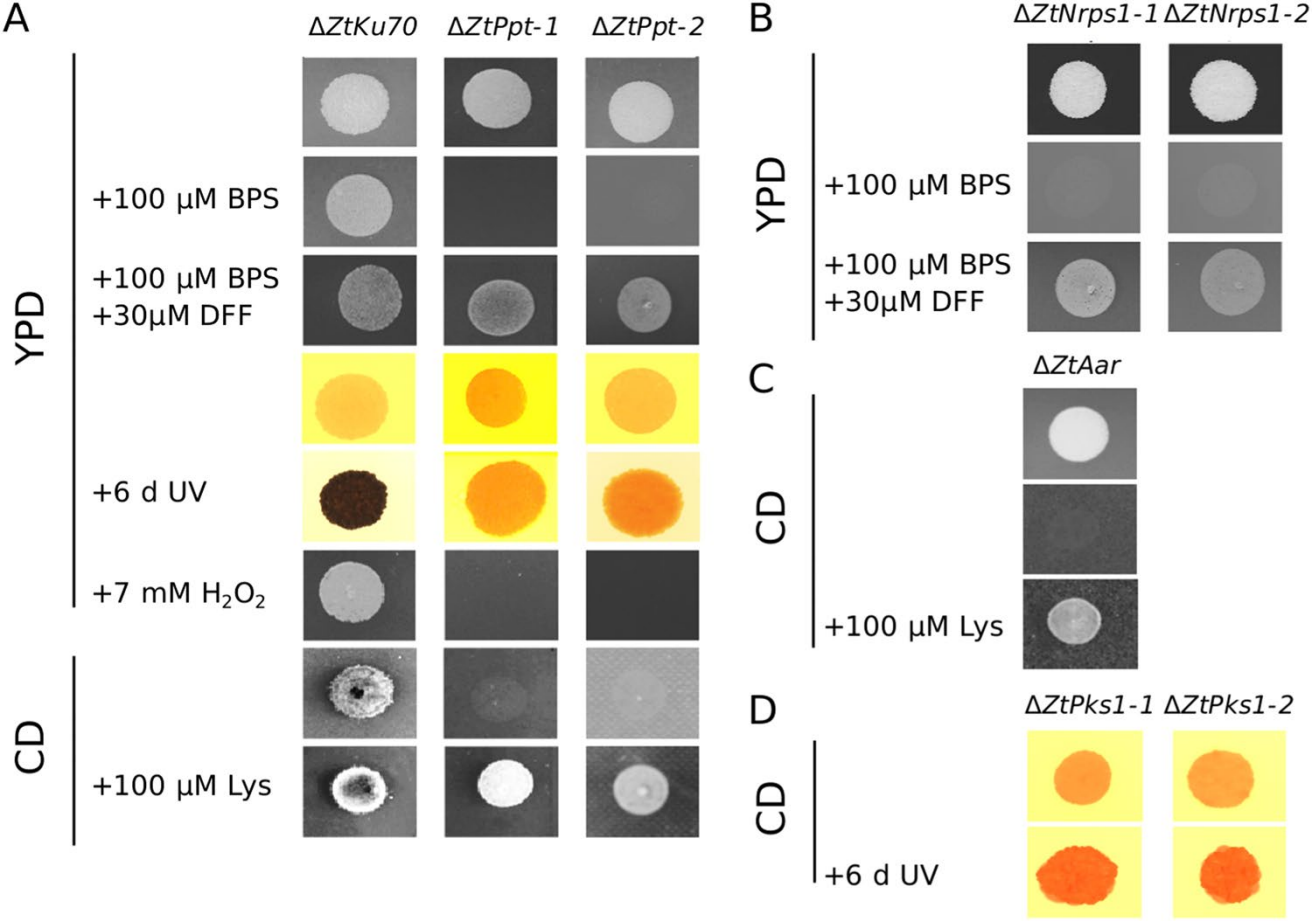
Functional characterisation of *∆ZtPpt* and related genes for auxotrophic and stress responses. (**A**) From top to bottom, WT (*ΔZtKu70*), *∆ZtPpt-1* and Δ*ZtPpt-2* grown on YPD agar, YPD agar with added BPS to remove available iron, YPD agar supplemented with the BPS and DFF to remove and then restore available iron, YPD agar grown in darkness, YPD agar after a further six days of exposure to UV light, YPD agar with the ROS species hydrogren peroxide added, CD minimal agar without lysine, CD minimal agar with lysine. (**B**) From top to bottom, the two strains Δ*ZtNrps1-1* and Δ*ZtNrps1-2* grown on YPD agar, YPD agar with added BPS to remove available iron and YPD agar with added BPS and DFF to remove then restore available iron. (**C**) From top to bottom, the ∆*ZtAar* strain grown on CD minimal agar with lysine and CD minimal agar without lysine. (**D**) From top to bottom, the two melanin synthetase strains *∆ZtPks1-1* and *∆ZtPks1-2* after six days of growth on YPD in darkness and a further six days of growth under UV light. All YPD plates were grown for six days in darkness apart from those that underwent an additional six days of exposure to UV light.

*ZtPpt*, *ZtPks1, ZtNrps1* and *ZtAar* gene deletion strains were all generated (Supplementary Figure 1) and firstly assessed for their responses to different stresses *in vitro*. This revealed that Δ*ZtPpt* strains were hypersensitive to iron depletion, as were the Δ*ZtNrps1* mutants (Fig. 1A,B). This was evident in the observation that neither of these strains grew in the presence of the iron chelating agent BPS but were rescued with further addition of an exogenous siderophore compound, desferriferrichrome (DFF). Δ*ZtPpt* strains were also auxotrophic for lysine biosynthesis, as was also observed for the AAR mutant Δ*ZtAar* (Fig. 1A,C). Finally, Δ*ZtPpt* strains were unable to melanise when exposed to UV light for six days or longer, a phenotype also observed for Δ*ZtPks1* (Fig. 1A,D). This same phenotype was also recently observed for *ZtStuA* mutants^31^ (Supplementary Figure 2A,B) highlighting that both StuA and Ppt functions are required for melanisation in this fungus. This was further supported by our additional data on ZtStuA, which demonstrated it to be a nuclear localised protein (Supplementary Figure 2C), and to positively influence the expression of several key genes for the DHN- melanin biosynthesis pathway, including the *Pks1* gene itself (Supplementary Figure 2B and Supplementary Table 3).

### *Zymoseptoria tritici* filamentous growth on nutrient limiting agar requires lysine biosynthesis but not Nrps1-derived siderophores or melanin

**Figure 2:**
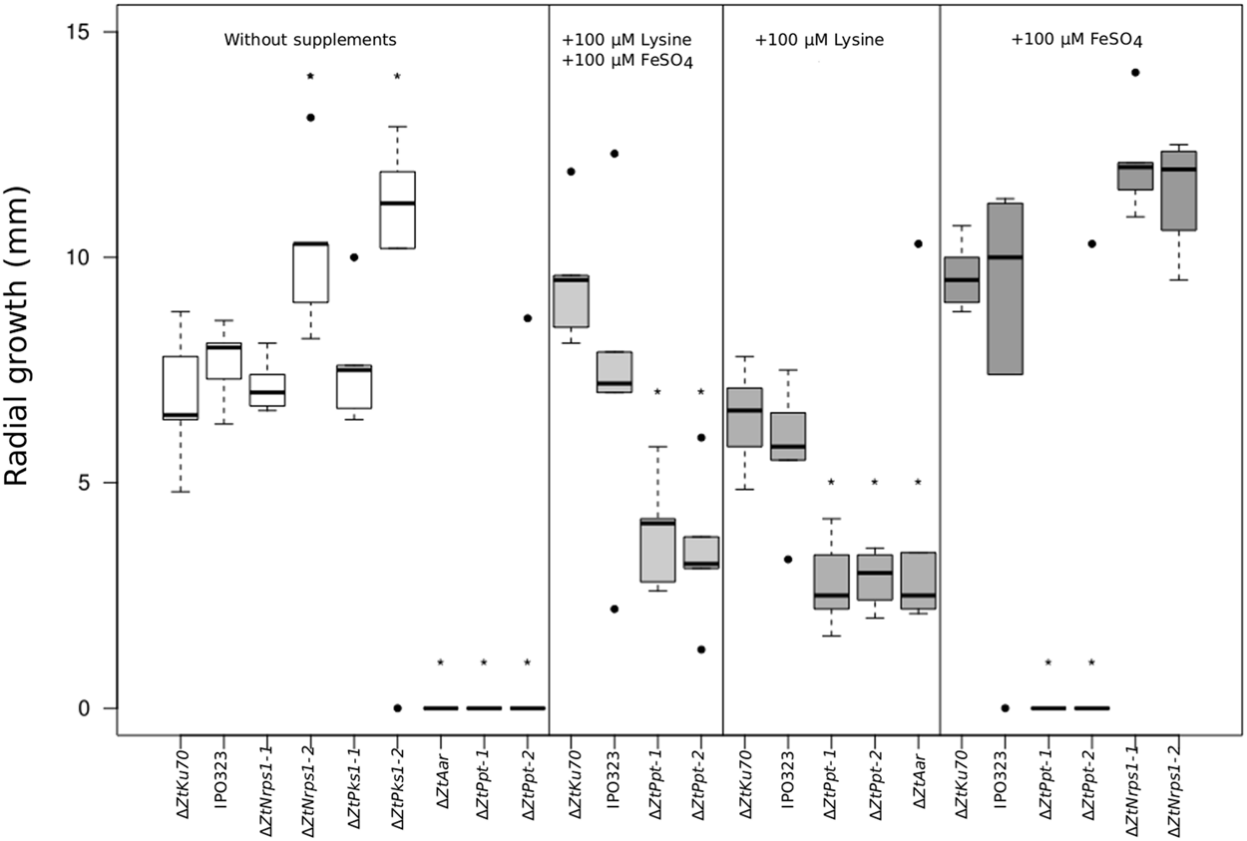
*In vitro* growth assays of Ppt-associated deletion strains on water agar with and without supplementation with lysine and iron. Measurement is radial growth after 20 days. From left to right portions of figure: the two WT strains and all PPT associated strains, ∆*ZtPpt-1*, ∆*ZtPpt-2*, ∆*ZtPks1-1*, ∆*ZtPks1-2*, ∆*ZtNrps1-1*, ∆*ZtNrps1-2* and ∆*ZtAar* on water agar with no supplements; the two PPT mutant strains ∆*ZtPpt-1* and ∆*ZtPpt-2* and the two WTs on water agar with both added available iron (FeSO_4_) and lysine; The lysine auxotrophic PPT associated strains ∆*ZtPpt-1*, ∆*ZtPpt-2* and ∆*ZtAar* and the two WTs on water agar supplemented with lysine; the siderophore mutant strains ∆*ZtNrps1-1* and ∆*ZtNrps1-2*, the PPT mutant strains ∆*ZtPpt-1* and, ∆*ZtPpt-2* and the two WTs on water agar supplemented with FeSO_4_. Horizontal black bars represent median values, boxes represent second and third quartiles and whiskers represent interquartile range. Asterisks represent significant differences in radial growth relative to the WT that constituted the background strain at α < 0.05.

To evaluate whether Δ*ZtPpt* strains were affected in filamentous growth *in vitro*, mutant strains were spot inoculated onto water agar, a nutrient depleted substrate which stimulates the formation of hyphal filaments from *Z. tritici* spores^34–36^. This showed that, in the absence of exogenous lysine, Δ*ZtPpt* and Δ*ZtAar* could not extend hyphal filaments (Fig. 2 and Supplementary Figure 3). When lysine was added, filamentous growth was rescued in these strains but not to wild type (WT) levels (Fig. 2 and Supplementary Figure 3). Both the Δ*ZtNrps1* and Δ*ZtPks1* strains grew to at least WT levels whether supplemented with lysine or not. One of each of the Δ*ZtNrps1* and Δ*ZtPks1* strains grew significantly faster than the WT without supplements, though this was not consistent across both strains assayed (Fig. 2).

### Lysine biosynthesis is a key requirement for *Z. tritici* infection of wheat but the Nrps1-derived siderophore and melanin are not

**Figure 3:**
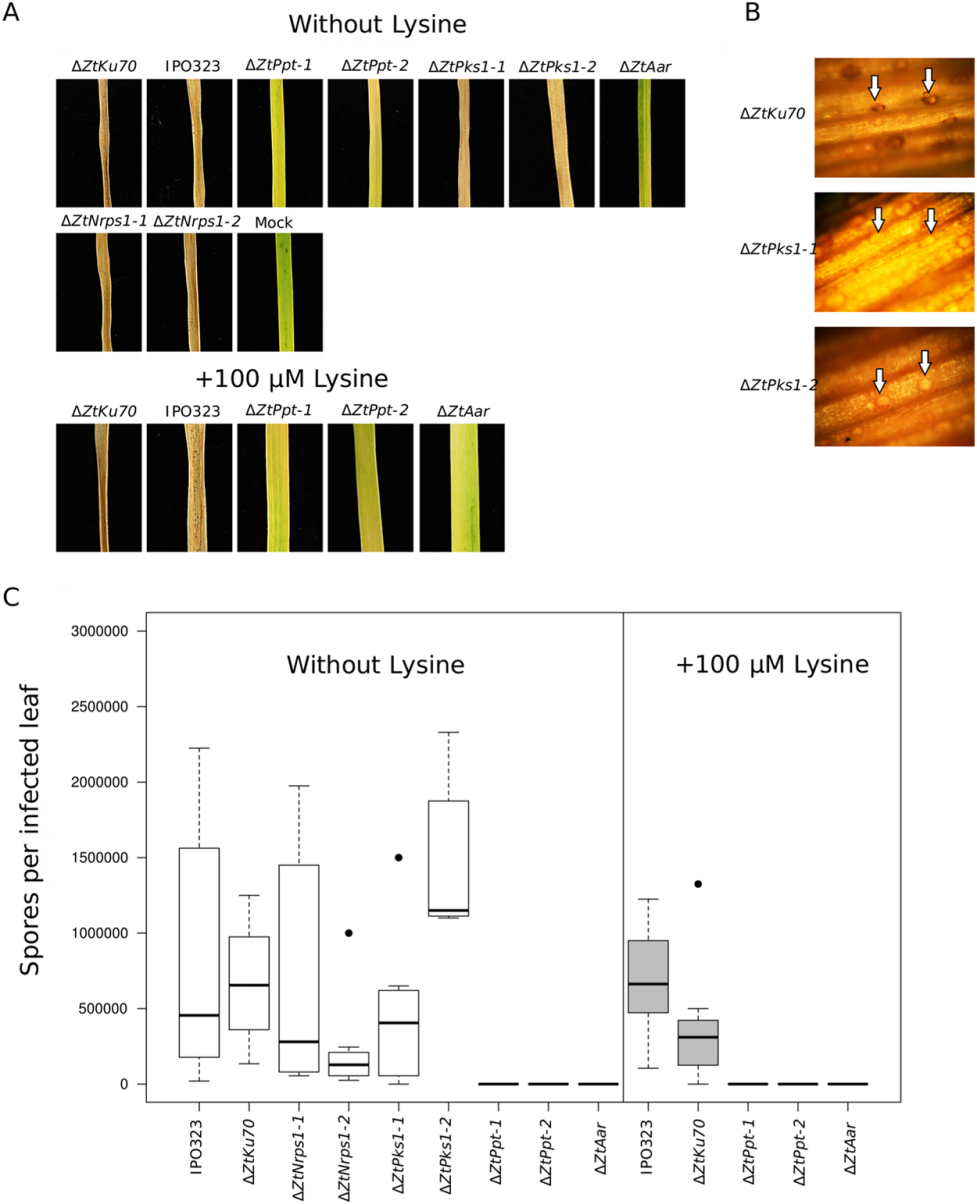
*In planta* inoculation assays for Ppt-associated gene deletion strains highlights the key role of lysine biosynthesis for virulence. (**A**) Upper panel: All PPT associated disruption strains, ∆*ZtPpt-1*, ∆*ZtPpt-2*, ∆*ZtPks1-1*, ∆*ZtPks1-2*, ∆*ZtNrps1-1*, ∆*ZtNrps1-2* and ∆*ZtAar*, and the two wild type strains IPO323 and ∆Z*tku70* after 22 days post inoculation (dpi) of the susceptible wheat cv, Riband; lower panel: the *Z. tritici* lysine auxotrophic PPT associated strains ∆*ZtPpt-1*, ∆*ZtPpt-2* and ∆*ZtAar*, and the two wildtype strains after 22 (dpi) with lysine added to starting inoculum. (**B**) The WT ∆Z*tku70*, and ∆*ZtPks1-1* and ∆*ZtPks1-2* melanin synthetase mutants viewed under a light microscope after 22 dpi. White arrows mark pycnidia, which in the melanin synthetase mutants are unpigmented. (**C**) Spores collected from infected leaves after 22 dpi. Left portion of plot: all PPT associated strains and the WTs without lysine added to fungal inoculum; right portion of plot: lysine auxotrophic PPT associated strains and the WTs with lysine added to fungal inoculum. Asterisks represent significant differences in spore counts relative to the WT that constituted the background strain (either IPO323 for Δ*ZtAar* or Δ*ZtKu70* for the rest) at α < 0.05. Black horizontal bars represent median values, boxes represent second and third quartiles and whiskers represent interquartile range.

We next assayed the virulence of Δ*ZtPpt* and the Δ*ZtPks1*, Δ*ZtNrps1* and Δ*ZtAar* downstream gene deletion mutants, on a susceptible wheat cultivar. This showed that Δ*ZtPpt* was completely avirulent both with and without lysine supplementation in the starting inoculum on wheat seedlings (Fig. 3A,C; Supplementary Figure 4). The same phenotype was also observed for the Δ*ZtAar* strain. In contrast, the Δ*ZtNrps1* strains were not affected in virulence, nor were the Δ*ZtPks1* strains (Fig. 3A,C). However, Δ*ZtPks1* generated non-melanised pycnidia at the end of the infection period (Fig. 3A,B). *ΔZtStuA* deletion mutants were recently shown to be attenuated in virulence on wheat^31^ and our data provided further support for this, which manifested itself in a delayed onset of leaf necrosis and lesions lacking asexual spores (Supplementary Figure 2D).

### Two additional *Zymoseptoria tritici PKS* genes strongly up-regulated during leaf infection are not required for virulence

**Figure 4:**
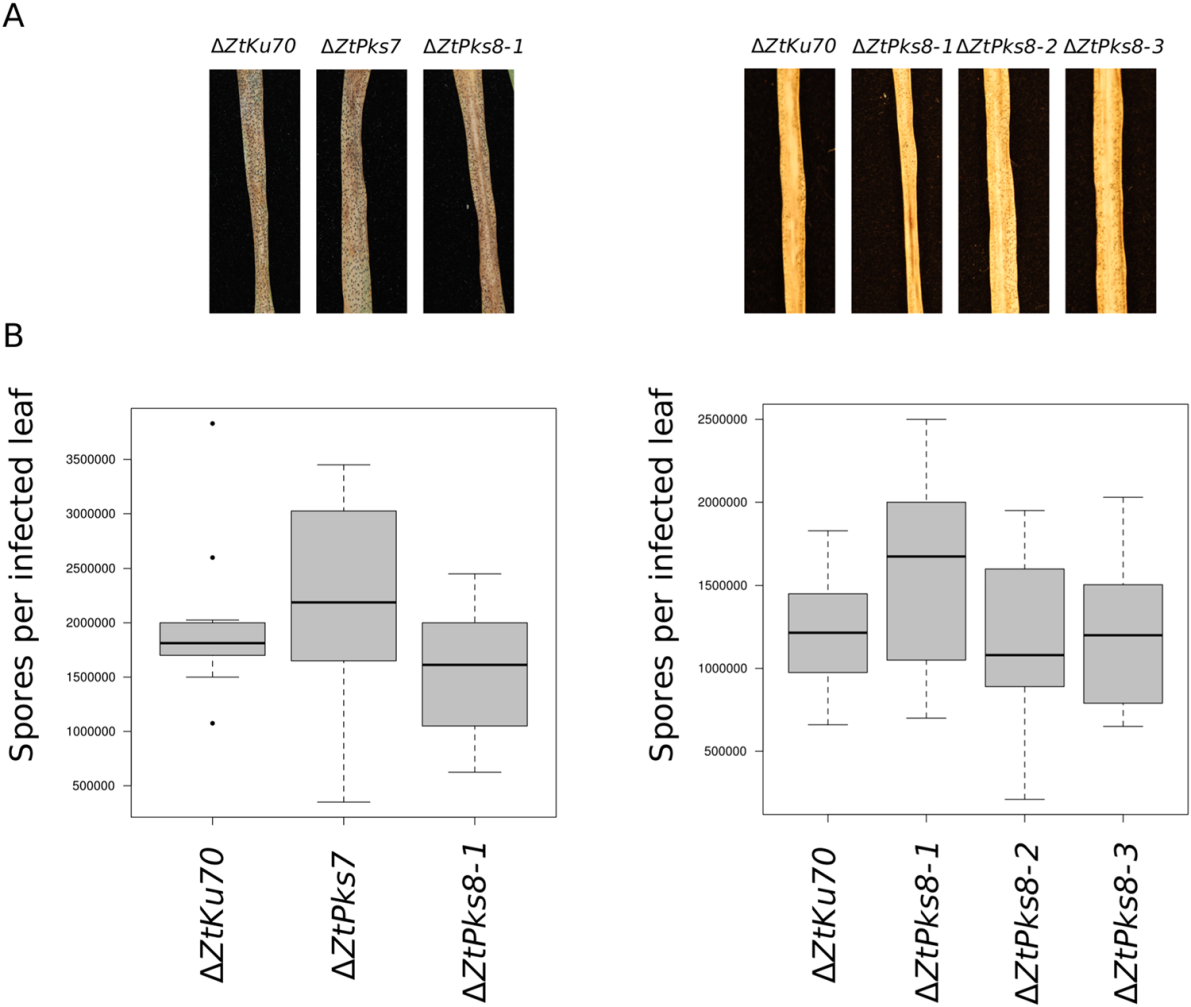
*In planta* inoculation assays for the two PKS deletion mutant strains ∆*ZtPks7* and ∆*ZtPks8* reveal them to be dispensable for virulence. (**A**) Symptoms after 22 dpi on the susceptible wheat cv. Riband. Left: The WT (∆Z*tKu70*), the sole ∆*ZtPks7* strain and one of the ∆*ZtPks8* strains, ∆*ZtPks8-1*; right: The WT (∆*Ztku70*) and three ∆*ZtPks8* mutant strains, ∆*ZtPks8-1*, ∆*ZtPks8-2* and ∆*ZtPks8-3*. (**B**) Spores retrieved from leaves after 22 dpi. Horizontal bars represent median values, boxes represent second and third quartiles and whiskers represent interquartile range. No significant differences were detected.

To assess the importance of two previously identified genes, *ZtPks7* and *ZtPks8*, whose gene clusters were transcriptionally up-regulated during infection^37^, deletion strains were generated and inoculated onto a susceptible wheat cultivar to test for altered virulence. For the single *∆ZtPks7* strain and the three ∆*ZtPks8* strains tested, no effects on virulence were observed (Fig. 4). After the full course of infection at 21 days, necrotic symptoms indistinguishable from the WT were observed in all cases (Fig. 4A). The number of spores retrieved from infected leaves for each of the mutant strains was also not significantly different to that for the WT strain (Fig. 4B,C).

## Discussion

**Figure 5:**
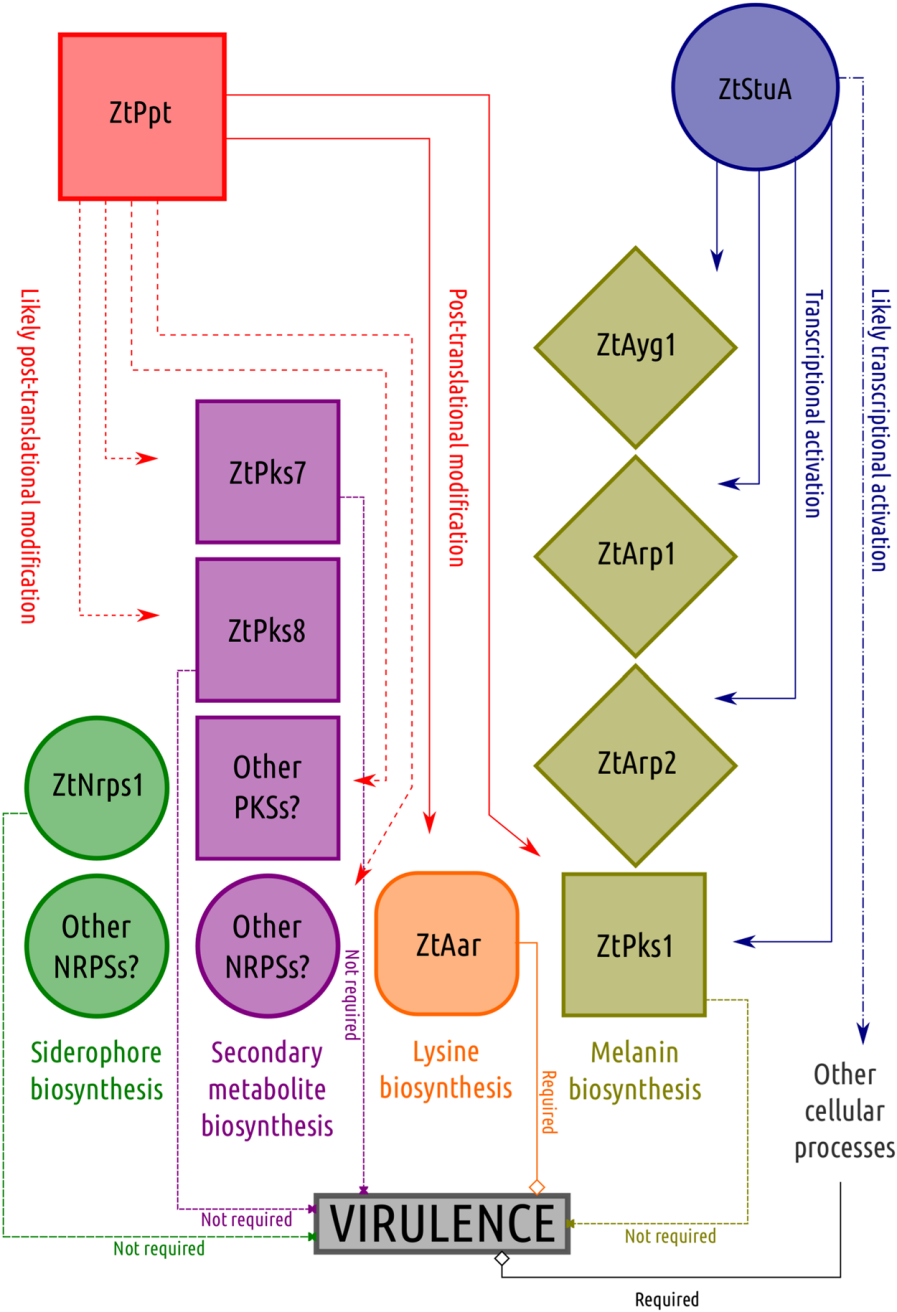
Summary of fungal knockout strains generated in this study, downstream processes regulated by them and effects on virulence.

In this study we analysed the relative importance of particular primary and secondary metabolites for virulence and other processes in the apoplastic wheat pathogen *Z. tritici*. We functionally characterised several *Z. tritici* genes, some of which were homologues of those known in other fungi to regulate lysine biosynthesis, melanisation and siderophore biosynthesis, amongst other processes. A summary overview of the deletion strains generated, the downstream processes they regulate, and their contributions (or not) to virulence is presented in Fig. 5. The schematic also incorporates data from the recently characterised StuA mutants of *Z. tritici*^31^ as well as our own novel insights presented here which demonstrated StuA to have a direct impact on the expression of PKS1 and other melanin pathway genes.

In agreement with prior studies^31^ *∆ZtStuA* mutants were defective in melanisation and we have now shown that the *Z. tritici* gene *ZtPks1*, was not expressed in this mutant, neither were other genes predicted to be in the melanin-biosynthesis pathway. *∆ZtPks1* deletion mutants themselves did not exhibit melanisation. These data together provide clear evidence that ZtPks1 is involved in melanin biosynthesis and ZtStuA can transcriptionally regulate it. That *ZtPks1* was involved in melanin biosynthesis has been suggested before by Cairns *et al*. (2017) and Lendenmann *et al*. (2015)^17,18^ but here we provide the experimental validation of this prediction. Interestingly, a recent QTL-based analysis of natural variation in the rate of melanisation by different *Z. tritici* strains has also revealed a second transcriptional regulator which positively influences *Pks1* expression, *Zmr1*^38^. This suggests that either StuA and Zmr1 exert their effects on *Pks1* and melanisation separately, or they may form part of, an as yet undescribed signalling pathway controlling *Pks1* expression. These two possibilities require further testing.

Alike the *∆ZtStuA* and *∆ZtPks1* mutants, *∆ZtPpt* mutants were also unable to melanise suggesting that the protein does indeed function to provide the essential co-factor for PKS and NRPS enzymes, including Pks1. This would indicate that the disrupted PPT gene functions similarly to those in other fungi to phosphopantetheinylate PKS and NRPS enzymes, including ZtPks1. ∆Z*tPpt* mutant strains were also hypersensitive to iron depletion and ROS, and auxotrophic for lysine. This further supports the idea that ZtPpt is a functional homologue of the PPT genes characterised in other fungi.

*ZtNrps1* mutant strains were hypersensitive to iron depletion, which would indicate that this gene encodes an enzyme that synthesises an important siderophore in a non-redundant manner under the conditions tested. Growth was restored when extracellular siderophores were added to the medium. In the model fungus *A. fumigatus* two major *NRPS* genes involved in siderophore biosynthesis have been characterised. These are *sidC*, which is required for biosynthesis of intracellular ferricrocin siderophores, and *sidD*, which is required for biosynthesis of extracellular fusarin C siderophores^39^. Intracellular siderophores are required for iron storage and transport, whereas extracellular siderophores are required for chelation and uptake of unavailable iron. *ZtNrps1* was more similar to sidD than sidC, supporting the hypothesis that it produces an extracellular siderophore. However, *ZtNrps1* was not necessary for wheat infection by *Z. tritici*. This is in contrast to previous reports on other fungi which have shown homologues of sidD (ZtNrps1) to be important for plant pathogenicity^40^. A notable exception to this was *Ustilago maydis*, where targeted disruption of biosynthesis of all siderophores in this fungus did not reduce virulence^41^. Since *U. maydis* is a biotrophic fungus and *Z. tritici* exhibits an extensive biotrophic/symptomless phase, it may be that the fungus has sufficient easily accessible iron, which overrides the need for extracellular siderophore production during leaf infection. Alternatively, *Z. tritici* may be able to use stored iron during filamentous growth. This is supported by the ability of *∆ZtNrps1* mutant strains to produce WT levels of hyphal filaments on iron-free water agar.

In contrast to the *∆ZtNrps1 and ∆ZtPks1* strains*, ∆ZtPpt* and ∆*ZtAar* mutants were unable to grow hyphal filaments *in vitro* or undergo budding growth on minimal medium without lysine supplementation. This indicates that both filamentous and budding growth in *Z. tritici* require a fully functioning lysine biosynthetic pathway. This is consistent with the lysine auxotrophy observed in Ppt mutant strains in various filamentous fungi studied to date^22,23,25^. The virulence data on wheat leaves demonstrated that both *∆ZtNrps1 and ∆ZtPks1* exhibited WT virulence, but both *∆ZtPpt* and ∆*ZtAar* were strongly reduced in virulence. Together this suggests that lysine availability (or lack of) forms a major barrier to infection by the latter two mutant strains, most probably as a result of restricting hyphal growth, and it is the loss of *ZtAar* function which is largely responsible for the loss of virulence in *∆ZtPpt*. Hence, both ZtPpt and ZtAar would appear to be good targets for putative fungicides for the control of STB.

In a recent study^37^ it was shown that *Z. tritici* exhibits differential expression of several other large putative SM clusters during infection. Several of these gene clusters contained PKSs highly upregulated coincident with the first appearance of necrotic lesions on wheat leaves. However, functional studies reported here have now shown that two of these PKSs are not essential for infection of the susceptible host cultivar tested, although we cannot rule out possible interactions with other cultivars. Ablation of individual PKS enzymes often has no discernible effect on virulence in plant pathogens. For example, in *Fusarium graminearum*, 15 PKS genes were disrupted in a single study and none affected virulence^42^. Hence, the functional roles of individual SM clusters in *Z. tritici* remain unknown. Besides their roles in host necrosis as toxins, SMs may be involved in, for example, microbial competition. In *T. reesei*, mutants for a specific PKS were less able to inhibit the growth of competing microbes, including the plant pathogens *Alterneria alternata, Sclerotinia sclerotiorum, Botrytis cinerea* and *Rhizoctonia solani*^43^. It is possible that *Z*. *tritici* specifically produces these energetically costly compounds, at a time when necrosis appears, to compete with other microbes that may wish to utilise the dead tissue. However, this, and the roles of several other predicted secondary metabolite clusters in the biology of *Z*. *trtici*, require further testing.

## Methods

### Strains, media and growth conditions

The fully sequenced *Z. tritici* reference strain IPO323^6^ and its derivative (Δ*ZtKu70*), in which the *Ku70* gene has been disrupted to reduce non-homologous end joining (NHEJ)^44^, were employed as wild type (WT) and recipient strains for gene deletion and gene disruption. Both strains are highly virulent on the susceptible wheat cultivars Taichung 29 and Riband. The WT and all generated strains were stored at −80 °C and re-cultured on potato dextrose agar (PDA) or yeast peptone dextrose (YPD) agar (Sigma-Aldrich Chemie, Steinheim, Germany) at 18 °C. Yeast-like spores were produced on V8 juice medium (Campbell Foods, Puurs, Belgium) or in yeast glucose broth (YGB) medium (yeast extract 10 g/L, glucose 20 g/L) placed in an orbital shaker (Innova 4430; New Brunswick Scientific, Nijmegen, The Netherlands) at 18 °C. To induce mycelial growth, all *Z. tritici* strains were grown under the same conditions but at 24 °C. To assess lysine auxotrophy, 100 mM Lysine was added to the minimal medium Czapek-Dox (CD) agar. To induce filamentous growth *in vitro* strains were grown on 10% sterile distilled water (SDW) agar.

### Bioinformatic analyses

Phylogenetic analysis of ZtStuA with homologues from other fungal pathogens was performed using the CLC genomics workbench package (Aarhus, Denmark). All StuA fungal proteins were retrieved from public databases and aligned with a gap opening cost and gap extension penalty of 10 and 1, respectively. The phylogenetic tree was constructed based on the unweighted pair group method with arithmetic average (UPGMA) algorithm, and support for the tree was assessed with 1000 bootstraps.

Additionally, the genome sequence of *Z. tritici* IPO323 (Goodwin *et al*., 2011) was queried in a BLAST search using the characterised sequences of PPT, NPS6 (a siderophore biosynthesetic enzyme), PKS1 (the fungal melanin biosynthetic enzyme) and AAR (the lysine biosynthesis enzyme) from *C. sativus* (Leng and Zhong, 2012). This was done using the BLASTp program over the NCBI BLAST server (https://blast.ncbi.nlm.nih.gov/Blast.cgi?PAGE = Proteins) with default settings. The PKS sequences *ZtPks7* and *ZtPks8* first identified as having a potential role in infection^37^ were also identified using the *Z. tritici* IPO323 genome sequence.

A further BLASTp analysis was conducted against the *Z. tritici* annotations using the *A. fumigatus* accessions XP_753088.1, XP_746662.1, XP_748660.1 and XP_748685.1, which correspond to the amino acid predictions for genes *sidC*, *sidD, sidF* and *sigG*, respectively. The former, *sidC* and *sidD*, encode NRPSs that produce intracellular ferricrocin and extracellular fusarin C siderophores, respectively. The latter, *sidF* and *sidG*, encode non-NRPS siderphore synthesis-associated enzymes^39^.

### Generation of gene replacement, complementation and GFP fusion constructs

To generate the *ZtStuA* deletion construct, pZtStuAKO, the USER friendly cloning method was used with minor modifications as described previously^45,46^. Briefly, ZtStuA-F3 and R3, as well as ZtStuA-F4 and R4 primer pairs were used to amplify about 2,000 bp upstream and downstream of ZtStuA using PfuTurbo® Cx Hotstart DNA polymerase (Stratagene, Cedar Creek, TX, US). In parallel, the pRF-HU2 vector possessing the *hygromycin phosphotransferase* (*hph*) gene as a selection marker was digested with two restriction enzymes, PacI and a nicking enzyme Nt.BbvCI, to generate a compatible overhang with the PCR amplicons. Subsequently, the PCR amplicons and the digested vector were mixed and treated with the USER enzyme mix (New England Biolabs, Ipswich, USA) and incubated at 37 °C for 30 min followed by 25 °C for 30 min. The resulting reaction was directly transformed into *Escherichia coli* strain DH5α and subsequently cultured on kanamycin-selective medium.

To identify bacterial colonies carrying the construct with the insertions in the expected positions, colony PCR was conducted using User-F and User-R primers (located in the middle of the *hph* gene) in combination with ZtStuA-F1 and ZtStuA-R1, respectively.

To produce the *ZtStuA* complementation construct and ZtStuA fused to GFP, the vectors pCZtStuA.com and pCZtStuA.GFP were generated by *in vivo* recombination in the yeast *Saccharomyces cerevisiae* DS94 (MATa, *ura3-52, trp1-1, leu2-3, his3-111*, and *lys2-801*)^47^ following published procedures^48,49^. The vector pCZtStuA.com contains the *ZtStuA* ORF under the control of the native promoter (1,025 bp) and terminator sequences (495 bp) for random ectopic integration into the genome of *Z. tritici* by using BASTA as the selection agent, while the vector pCZtStuA.GFP contains ZtStuA.GFP fused to the full-length *ZtStuA* under the control of the constitutive *ZtTub2* promoter and terminator sequences for targeted integration into the *sdi1* locus of *Z. tritici* by using carboxin as the selection agent. A 9,760 bp fragment of pCGEN-YR (digested with *Xba*I and *Zra*I^48^), 1410 bp fragment covering the BASTA resistance cassette *bar* (amplified with MG-Sep-106 and MG-Sep-107), 3146 bp fragment covering the 1025 bp of *ZtStuA* promoter, 1626 bp pf *ZtStuA* gene and 495 bp of *ZtStuA* terminator (amplified with MG-Sep-113 and MG-Sep-116) were recombined in yeast *S. cerevisiae* to obtain the vector pCZtStuA.com. A 16,597 bp fragment of pCZtGFPSpa2 (digested with *Psh*AI^50^), 1626 bp full-length *ZtStuA* gene (amplified with SK-Sep-346 and SK-Sep-347) were recombined in yeast *S. cerevisiae* to obtain the vector pCZtGFPStuA.

To produce the disruption constructs for the Ppt-associated genes *ZtNrps1* and *ZtPks1*, and the PKS genes *ZtPks7* and *ZtPks8*, primers with added 5′ restriction sites and two 5′ adenines were designed for the amplification of the selected flanking sequences using Geneious version 8.1. Flanking sequences were approximately 1 Kb either side of these genes. Flanking regions were amplified in PCR reactions from genomic DNA of isolate IPO323 using RedTaq mastermix according to the manufacturer’s protocol. Thermocycler settings were 95 °C 2 min, then 30 cycles of 95 °C 30 sec, 60 °C 30 sec, 72  °C 2 min, and 10 °C hold. Flanking sequences were cloned into pCHYG^51^ either side of *hph* under the *trpC* promoter. For all strains generated in this study, primers and added restriction sites are in Supplementary Table 4.

The ∆*ZtAar* strain was generated in Ali *et al*.^52^. Briefly, pCambia 0380 was digested with SacII, the 2-micron + ura3 region was amplified by PCR from pYES2 using 2 µmURA3 FP and 2 µmURA3 RP was used to repair the plasmid by in-yeast recombination. The left and right flanks for *ZtAar* were amplified from genomic DNA by PCR and recombined into BamH1-digested pCambia0380YA along with the hygromycin resistance cassette. Plasmids were purified from yeast using the Zymoprep ITM yeast plasmid extraction kit and rescued into *E. coli* then construction confirmed by restriction digest, PCR and sequencing across recombination sites.

### Fungal transformation

The gene deletion construct as well as the replacement construct were first cloned into *A. tumefaciens* strain AGL1 via electroporation. *Agrobacterium* mediated transformation was then carried out to delete *ZtStuA* in the Δ*ZtKu70* strain as previously described^51^. For the complementation assay, the same procedure was applied but the putatively complemented strains were selected on plates with 50 µg mL^−1^ BASTA. All disrupted PKS and Ppt-associated mutant strains except Δ*ZtAar* were also generated in the Δ*ZtKu70* background^44^. Δ*ZtAar* was disrupted in a wild type IPO323 background^52^. For the Ppt and Aar mutants, 100 µM Lysine was added to the selection plates to ensure survival of transformed cells, which were expected to be lysine auxotrophs. Emerging colonies were then subjected to two rounds of selection on hygromycin amended YPD agar plates. Genomic DNA of stable transformants was extracted and used in PCR screens to confirm homologous recombination in the correct positions.

### Epi-fluorescence microscopy

Fluorescence microscopy was performed as previously described^53^. In brief, IPO323_CZtGFPStuA cells were inoculated in YG media and grown at either 18 °C with 200 rpm to induce the yeast-like cell growth or 24 °C with 100 rpm to induce the hyphal growth for 24 h and placed onto a 2% agar cushion for direct observation using a motorized inverted microscope (IX81; Olympus, Hamburg, Germany), equipped with a PlanApo 100_/1.45 Oil TIRF objective (Olympus, Hamburg, Germany). Fluorescent tags and dyes were exited using a VS-LMS4 Laser Merge System with solid-state lasers (488 nm/50 mW or 75 mW and 561 nm/50 mW or 75 mW; VisitronSystems, Puchheim, Germany) and single images or z-Stacks, using an objective piezo (Piezosystem Jena GmbH, Jena, Germany), over 6 µm depth with a z resolution of 0.2 µm were captured with150 ms exposure. In addition a DIC image was taken for each cell using a CoolSNAP HQ2 camera (Photometrics/Roper Scientific,Tucson, USA). Overlays of the fluorescent and DIC images were generated using MetaMorph (Molecular Devices, Wokingham, UK). All parts of the system were under the control of the software package MetaMorph (MolecularDevices, Wokingham, UK). The localisation of ZtGFPStuA in the nucleus was confirmed by counterstaining the nucleus with DAPI (Sigma-Aldrich Company Ltd. Dorset, England). To this end, cells of strain IPO323_CZtGFPStuA were grown for 24 h in YG medium at 18 °C with 200 rpm. DAPI was added the cell culture to a final concentration of 1 µg/ml and incubated for 10 minutes at 18 °C with 200 rpm followed by direct observation using a motorized inverted microscope (IX81; Olympus, Hamburg, Germany). The DAPI as well as the ZtGFP were exited using a standard mercury burner and imaged at 500 ms exposure time.

### RNA isolation and q-RT-PCR

*In vitro* and *in planta* expression profiling of *ZtStuA* was performed using quantitative real-time PCR (q-RT-PCR). For *in planta* analyses, the wheat cv. Taichung 29 was inoculated with Δ*ZtKu70* as described previously^46^ and leaf samples were collected in three biological replicates, flash frozen and ground in liquid nitrogen using a mortar and pestle. Total RNA was extracted either from ground leaves or fungal biomass produced in YGB using the RNeasy plant mini kit (Qiagen, location, USA) and subsequently DNA contamination was removed using the DNAfree kit (Ambion, Cambridgeshire, U.K.). First-strand cDNA was synthesized from approximately two µg of total RNA primed with oligo(dT) using the SuperScript III according to the manufacturer’s instructions. One µl of the resulting cDNA was used in a 25 µl PCR reaction using a QuantiTect SYBR Green PCR Kit and run and analyzed using an ABI 7500 Real-Time PCR System. The relative expression of each gene was initially normalized to the constitutively expressed *Z. tritici β-tubulin* gene^54,55^ and then calculated based on the comparative C(t) method described previously^56^. Primers used in the expression profiling analysis are detailed in Supplementary Table 5.

### Analysis of sensitivity to different stressors *in vitro*

To identify *in vitro* defects caused by the disrupted PPT-associated genes, all mutant strains were 10 μl spot-inoculated from 10^6^ spores per ml suspensions in SDW onto YPD and/or CD minimal medium agar plates either with or without supplementation or incubation under UV light. To assess for increased ROS sensitivity, YPD plates were supplemented with 7 mM H_2_O_2_; to assess for melanisation deficiency YPD plates were incubated after an initial 6 day period for a further 6 days under UV light at 20 °C; to assess for lysine auxotrophy CD plates were supplemented with 100 μM lysine; and to assess for hypersensitivity to iron depletion YPD plates were supplemented with 100 μM bathophenanthroline disulphate (BPS) or 100 μM BPS and 30 μM desferriferrichrome (DFF). BPS is an iron chelating agent that reduces iron availability whereas DFF, a siderophore purified from *U. sphaerogena*, is an iron chelating agent that restores iron availability.

When placed onto a medium containing zero nutrients such as water agar, *Z. tritici* spores undergo filamentous growth. This contrasts the budding growth observed on nutrient replete media such as YPD agar. To assess defects in filamentous growth, the two WT controls and all targeted disruption strains were spot inoculated onto water agar as described in the previous paragraph. Water agar plates were either amended with 100 µM lysine, 100 µM FeSO_4_, a combination of both or neither. This was to assess the impact of different PPT-associated phenotypes on filamentous growth. After 20 days, radial growth of spot inoculations was measured vertically and horizontally and the mean value of these measurements was taken.

### Statistical analysis of infection assays and *in vitro* stress response tests for the Ppt-associated and Pks mutant strains

R version 3.2.3 was used for all statistical tests. For plant infection assays with the Ppt-associated and PKS strains, individual leaves if different seedlings were treated as replicates. Spore count data for leaves inoculated with each independent strain were assessed visually for how well they fit a normal distribution using quantile quantile plots and statistically using the Shapiro test for normality (Supplementary Figure 4). Based on data distributions observed, a log transformation was applied to each sample dataset before further analyses for Ppt associated strains (Supplementary Figure 4). One-way analysis of variance (ANOVA) was carried out on the transformed data, followed by Tukey’s honest significant difference test for pairwise differences. Differences were considered significant at α < 0.05. The analysis was carried out separately for the Δ*ZtPpt* (PPT), Δ*ZtPks1*, Δ*ZtNrps1* and Δ*ZtAar*, and the PKS mutants Δ*ZtPks7*, Δ*ZtPks8*. For the latter, no data transformations were applied as data mostly fit a normal distribution (Supplementary Figure 5b). Two independent strains (in one case three) were assessed for all but two disruptions, Δ*ZtAar* and Δ*ZtPks7*. The same statistical test was carried out for the radial filamentous growth data from the spot inoculations of water agar. In this instance data were not transformed as they were adequately normally distributed for ANOVA (Supplementary Figure 3).

### Pathogenicity assays

The susceptible wheat cultivars Taichung 29 and Riband were grown in a greenhouse until the first leaves were fully unfolded or for 17 days, respectively. Inoculum of all strains was produced in YGB (yeast extract 10 g/L, Glucose 30 g/L) at 18 °C for 7 days in an orbital shaker (Innova 4430; New Brunswick Scientific, Nijmegen, The Netherlands) and yeast-like spores were obtained after centrifugation at 3000 rpm and two washing steps to remove residual medium. Subsequently, the spore concentrations were adjusted to either 10^7^ spores mL^−1^ for the Δ*ZtStuA* assay or 10^6^ spores mL^−1^ for the infection assay of all other mutant strains. The resulting suspension was supplemented with 0.15% Tween 20 as a surfactant. Infection assays and counting of yeast-like cells on the infected leaves were conducted as previously described^46,57^.

## Electronic supplementary material

Supplementary information

## Data Availability

All strains generated in this study are available upon request from the corresponding authors.

## References

[1] Quaedvlieg W (2011). *Zymoseptoria* gen. nov.: a new genus to accommodate Septoria-like species occurring on graminicolous hosts. Persoonia.

[2] Eyal, Z. The septoria tritici and stagonospora nodorum blotch diseases of wheat. *European Journal of Plant Pathology* **105** (1999).

[3] Fones H, Gurr S (2015). The impact of Septoria tritici Blotch disease on wheat: An EU perspective. Fungal Genet. Biol..

[4] Torriani SFF (2015). *Zymoseptoria tritici*: A major threat to wheat production, integrated approaches to control. Fungal Genet. Biol..

[5] Hayes LE, Sackett KE, Anderson NP, Flowers MD, Mundt CC (2016). Evidence of Selection for Fungicide Resistance in *Zymoseptoria tritici* Populations on Wheat in Western Oregon. Plant Dis..

[6] Goodwin SB (2011). Finished genome of the fungal wheat pathogen *Mycosphaerella graminicola* reveals dispensome structure, chromosome plasticity, and stealth pathogenesis. PLoS Genet..

[7] Cools HJ, Hammond-Kosack KE (2013). Exploitation of genomics in fungicide research: current status and future perspectives. Mol. Plant Pathol..

[8] Kema G (1996). Histology of the Pathogenesis of *Mycosphaerella graminicola* in Wheat. Phytopathology.

[9] Markham JE, Hille J (2001). Host-selective toxins as agents of cell death in plant-fungus interactions. Mol. Plant Pathol..

[10] Lorang JM, Sweat TA, Wolpert TJ (2007). Plant disease susceptibility conferred by a “resistance” gene. Proc. Natl. Acad. Sci..

[11] Inderbitzin P, Asvarak T, Turgeon BG (2010). Six new genes required for production of T-toxin, a polyketide determinant of high virulence of *Cochliobolus heterostrophus* to maize. Mol. Plant. Microbe. Interact..

[12] Dewey RE, Siedow JN, Timothy DH, Levings CS (1988). A 13-kilodalton maize mitochondrial protein in *E. coli* confers sensitivity to Bipolaris *maydis toxin*. Science.

[13] Stergiopoulos I, Collemare J, Mehrabi R, De Wit PJGM (2013). Phytotoxic secondary metabolites and peptides produced by plant pathogenic Dothideomycete fungi. FEMS Microbiol. Rev..

[14] Chen L-H, Yang SL, Chung K-R (2014). Resistance to oxidative stress via regulating siderophore-mediated iron acquisition by the citrus fungal pathogen *Alternaria alternata*. Microbiology.

[15] Howard, R. J., Ferrari, A. A., Howard, R. J. & Ferrari, A. Role of Melanin in Appressorium Function. *Experimental Mycology* **13** (1989).

[16] Jiang H (2017). Both a PKS and a PPTase are involved in melanin biosynthesis and regulation of *Aureobasidium melanogenum* XJ5-1 isolated from the Taklimakan desert. Gene.

[17] Cairns T, Meyer V (2017). *In silico* prediction and characterization of secondary metabolite biosynthetic gene clusters in the wheat pathogen *Zymoseptoria tritici*. BMC Genomics.

[18] Lendenmann MH, Croll D, Stewart EL, McDonald BA (2014). Quantitative Trait Locus Mapping of Melanization in the Plant Pathogenic Fungus *Zymoseptoria tritici*. G3 Genes|Genomes|Genetics.

[19] Keszenman-Pereyra D, Lawrence S, Twfieg M-E, Price J, Turner G (2003). The npgA/cfwA gene encodes a putative 4′-phosphopantetheinyl transferase which is essential for penicillin biosynthesis in *Aspergillus nidulans*. Curr. Genet..

[20] Márquez-Fernández O (2007). Phosphopantetheinyl transferase CfwA/NpgA is required for *Aspergillus nidulans* secondary metabolism and asexual development. Eukaryot. Cell.

[21] Ehmann DE, Gehring AM, Walsh CT (1999). Lysine biosynthesis in Saccharomyces cerevisiae: mechanism of alpha-aminoadipate reductase (Lys2) involves posttranslational phosphopantetheinylation by Lys5. Biochemistry.

[22] Leng Y, Zhong S (2012). Sfp-type 4′-phosphopantetheinyl transferase is required for lysine synthesis, tolerance to oxidative stress and virulence in the plant pathogenic fungus Cochliobolus sativus. Mol. Plant Pathol..

[23] Horbach R (2009). Sfp-type 4′-phosphopantetheinyl transferase is indispensable for fungal pathogenicity. Plant Cell.

[24] Wiemann P (2012). The Sfp-type 4′-phosphopantetheinyl transferase Ppt1 of *Fusarium fujikuroi* controls development, secondary metabolism and pathogenicity. PLoS One.

[25] Velazquez-Robledo R (2011). Role of the 4-phosphopantetheinyl transferase of *Trichoderma virens* in secondary metabolism and induction of plant defense responses. Mol. Plant. Microbe. Interact..

[26] Dobb KS (2015). Characterisation of the *Candida albicans* Phosphopantetheinyl Transferase Ppt2 as a Potential Antifungal Drug Target. PLoS One.

[27] Johns A (2017). A Nonredundant Phosphopantetheinyl Transferase, PptA, Is a Novel Antifungal Target That Directs Secondary Metabolite, Siderophore, and Lysine Biosynthesis in *Aspergillus fumigatus* and Is Critical for Pathogenicity. MBio.

[28] Lee W-S, Rudd JJ, Hammond-Kosack KE, Kanyuka K (2014). *Mycosphaerella graminicola* LysM effector-mediated stealth pathogenesis subverts recognition through both CERK1 and CEBiP homologues in wheat. Mol. Plant. Microbe. Interact..

[29] Saintenac C (2018). Wheat receptor-kinase-like protein Stb6 controls gene-for-gene resistance to fungal pathogen *Zymoseptoria tritici*. Nat. Genet..

[30] Zhong Z (2017). A small secreted protein in *Zymoseptoria tritici* is responsible for avirulence on wheat cultivars carrying the Stb6 resistance gene. New Phytol..

[31] Tiley A, Bailey A, Foster G (2018). Exploring the genetic regulation of asexual sporulation in *Zymoseptoria tritici*. Front. Microbiol..

[32] Palma-Guerrero J (2017). Comparative Transcriptome Analyses in *Zymoseptoria tritici* Reveal Significant Differences in Gene Expression Among Strains During Plant Infection. Mol. Plant-Microbe Interact..

[33] Rudd Jason J., Kanyuka Kostya, Hassani-Pak Keywan, Derbyshire Mark, Andongabo Ambrose, Devonshire Jean, Lysenko Artem, Saqi Mansoor, Desai Nalini M., Powers Stephen J., Hooper Juliet, Ambroso Linda, Bharti Arvind, Farmer Andrew, Hammond-Kosack Kim E., Dietrich Robert A., Courbot Mikael (2015). Transcriptome and Metabolite Profiling of the Infection Cycle of Zymoseptoria tritici on Wheat Reveals a Biphasic Interaction with Plant Immunity Involving Differential Pathogen Chromosomal Contributions and a Variation on the Hemibiotrophic Lifestyle Definition. Plant Physiology.

[34] Mehrabi R, Zwiers L-H, de Waard MA, Kema GHJ (2006). *MgHog1* Regulates Dimorphism and Pathogenicity in the Fungal Wheat Pathogen *Mycosphaerella graminicola*. Mol. Plant-Microbe Interact..

[35] Motteram J (2011). Aberrant protein N-glycosylation impacts upon infection-related growth transitions of the haploid plant-pathogenic fungus *Mycosphaerella graminicola*. Mol. Microbiol..

[36] King R (2017). A conserved fungal glycosyltransferase facilitates pathogenesis of plants by enabling hyphal growth on solid surfaces. PLOS Pathog..

[37] Rudd JJ (2015). Transcriptome and metabolite profiling of the infection cycle of *Zymoseptoria tritici* on wheat reveals a biphasic interaction with plant immunity involving differential pathogen chromosomal contributions and a variation on the hemibiotrophic lifestyle definition. Plant Physiol..

[38] Krishnan P (2018). Transposable element insertions shape gene regulation and melanin production in a fungal pathogen of wheat. BMC Biol..

[39] Schrettl M (2007). Distinct roles for intra- and extracellular siderophores during *Aspergillus fumigatus* infection. PLoS Pathog..

[40] Oide S (2006). NPS6, encoding a nonribosomal peptide synthetase involved in siderophore-mediated iron metabolism, is a conserved virulence determinant of plant pathogenic ascomycetes. Plant Cell.

[41] Mei B, Budde AD, Leong SA (1993). sid1, a gene initiating siderophore biosynthesis in *Ustilago maydis*: molecular characterization, regulation by iron, and role in phytopathogenicity. Proc. Natl. Acad. Sci. USA.

[42] Gaffoor I (2005). Functional analysis of the polyketide synthase genes in the filamentous fungus *Gibberella zeae* (anamorph *Fusarium graminearum*). Eukaryot. Cell.

[43] Atanasova L, Knox BP, Kubicek CP, Druzhinina IS, Baker SE (2013). The Polyketide Synthase Gene *pks4* of *Trichoderma reesei* Provides Pigmentation and Stress Resistance. Eukaryot. Cell.

[44] Bowler J (2010). New capabilities for *Mycosphaerella graminicola* research. Mol. Plant Pathol..

[45] Frandsen RJN, Andersson JA, Kristensen MB, Giese H (2008). Efficient four fragment cloning for the construction of vectors for targeted gene replacement in filamentous fungi. BMC Mol. Biol..

[46] Mirzadi Gohari A (2014). Molecular characterization and functional analyses of ZtWor1, a transcriptional regulator of the fungal wheat pathogen *Zymoseptoria tritici*. Mol. Plant Pathol..

[47] Tang X, Halleck MS, Schlegel RA, Williamson P (1996). A subfamily of P-type ATPases with aminophospholipid transporting activity. Science.

[48] Kilaru S, Steinberg G (2015). Yeast recombination-based cloning as an efficient way of constructing vectors for *Zymoseptoria tritici*. Fungal Genet. Biol..

[49] Raymond, C. K., Pownder, T. A. & Sexson, S. L. General method for plasmid construction using homologous recombination. *Biotechniques* **26**, 134–8, 140–1 (1999). 10.2144/99261rr02 9894602

[50] Guo M, Kilaru S, Schuster M, Latz M, Steinberg G (2015). Fluorescent markers for the Spitzenkörper and exocytosis in Zymoseptoria *tritici*. Fungal Genet. Biol..

[51] Zwiers LH, De Waard MA (2001). Efficient *Agrobacterium tumefaciens*-mediated gene disruption in the phytopathogen *Mycosphaerella graminicola*. Curr. Genet..

[52] Ali, S. J. Investigating secondary metabolism in *Zymoseptoria tritici*. (2015).

[53] Kilaru S (2015). A gene locus for targeted ectopic gene integration in *Zymoseptoria tritici*. Fungal Genet. Biol..

[54] Keon J (2007). Transcriptional adaptation of *Mycosphaerella graminicola* to programmed cell death (PCD) of its susceptible wheat host. Mol. Plant. Microbe. Interact..

[55] Motteram J (2009). Molecular Characterization and Functional Analysis of *MgNLP*, the Sole NPP1 Domain–Containing Protein, from the Fungal Wheat Leaf Pathogen *Mycosphaerella graminicola*. Mol. Plant-Microbe Interact..

[56] Schmittgen TD, Livak KJ (2008). Analyzing real-time PCR data by the comparative C(T) method. Nat. Protoc..

[57] Derbyshire, M. C. *et al*. Analysis of cytochrome b< inf >5</inf >reductase-mediated metabolism in the phytopathogenic fungus Zymoseptoria tritici reveals novel functionalities implicated in virulence. *Fungal Genet. Biol*. **82** (2015). 10.1016/j.fgb.2015.05.008 PMC4557397 26074495

