# Author Correction: Phosphopantetheinyl transferase (Ppt)-mediated biosynthesis of lysine, but not siderophores or DHN melanin, is required for virulence of *Zymoseptoria tritici* on wheat

**DOI:** 10.1038/s41598-019-53309-9

**Published:** 2019-11-07

**Authors:** Mark C. Derbyshire, Amir Mirzadi Gohari, Rahim Mehrabi, Sreedhar Kilaru, Gero Steinberg, Solaf Ali, Andy Bailey, Kim Hammond-Kosack, Gert H. J. Kema, Jason J. Rudd

**Affiliations:** 1 BioIntercations and Crop Protection, Rothamsted Research, Harpenden, Hertfordshire, UK; 2 Centre for Crop and Disease Management, Curtin University, Perth, Australia; 3 Department of Plant Pathology, Faculty of Agricultural Sciences and Engineering, College of Agriculture and Natural Resources, University of Tehran, Karaj, Iran; 4 Wageningen University and Research, Wageningen Plant Research, PO Box 16, 6700AA Wageningen, The Netherlands; 5 Department of Biotechnology, College of Agriculture, Isfahan University of Technology, Isfahan, 84156-83111 Iran; 6 Biosciences, University of Exeter, Exeter, EX4 4QD UK; 7 Technical College of Health, Sulaimani Polytechnic University, Qrga, Wrme Street, Mardin 327, Alley 76, Sulaimaniyah, Kurdistan Region of Iraq, Sulaimani Governorate, Iraq; 8 Wageningen University and Research, Laboratory of Phytopathology, PO box 16, 6700AA Wageningen, The Netherlands

Correction to: *Scientific Reports* 10.1038/s41598-018-35223-8, published online 20 November 2018

In the original version of this Article, Solaf Ali was incorrectly affiliated with ‘School of Biological Sciences, Bristol University, 24 Tyndall Avenue, Bristol, UK’. The correct affiliation is listed below.

Technical College of Health, Sulaimani Polytechnic University, Qrga, Wrme Street, Mardin 327, Alley 76, Sulaimaniyah, Kurdistan Region of Iraq, Sulaimani Governorate, Iraq.

This error has now been corrected in the PDF and HTML versions of the Article.

