# Supplementary material for: Phosphopantetheinyl transferase (Ppt)-mediated biosynthesis of lysine, but not siderophores or DHN melanin, is required for virulence of Zymoseptoria tritici on wheat
Source: Sci Rep. 2018 Nov 20;8:17069. doi: 10.1038/s41598-018-35223-8 (PMC6244202; doi:10.1038/s41598-018-35223-8)
Supplement: Supplementary file 1 — Supplementary information [file 41598_2018_35223_MOESM1_ESM.pdf]

Phosphopantetheinyl transferase (Ppt)-mediated biosynthesis of lysine, nut not siderophore or DHN melanin, is required for virulence of Zymoseptoria tritici on wheat.

Mark C Derbyshire, Amir Mirzadi Gohari, Rahim Mehrabi, Sreedhar Kilaru, Gero Steinberg, Solaf Ali, Andy Bailey, Kim Hammond-Kosack, Gert HJ Kema, Jason J Rudd

Supplementary material.

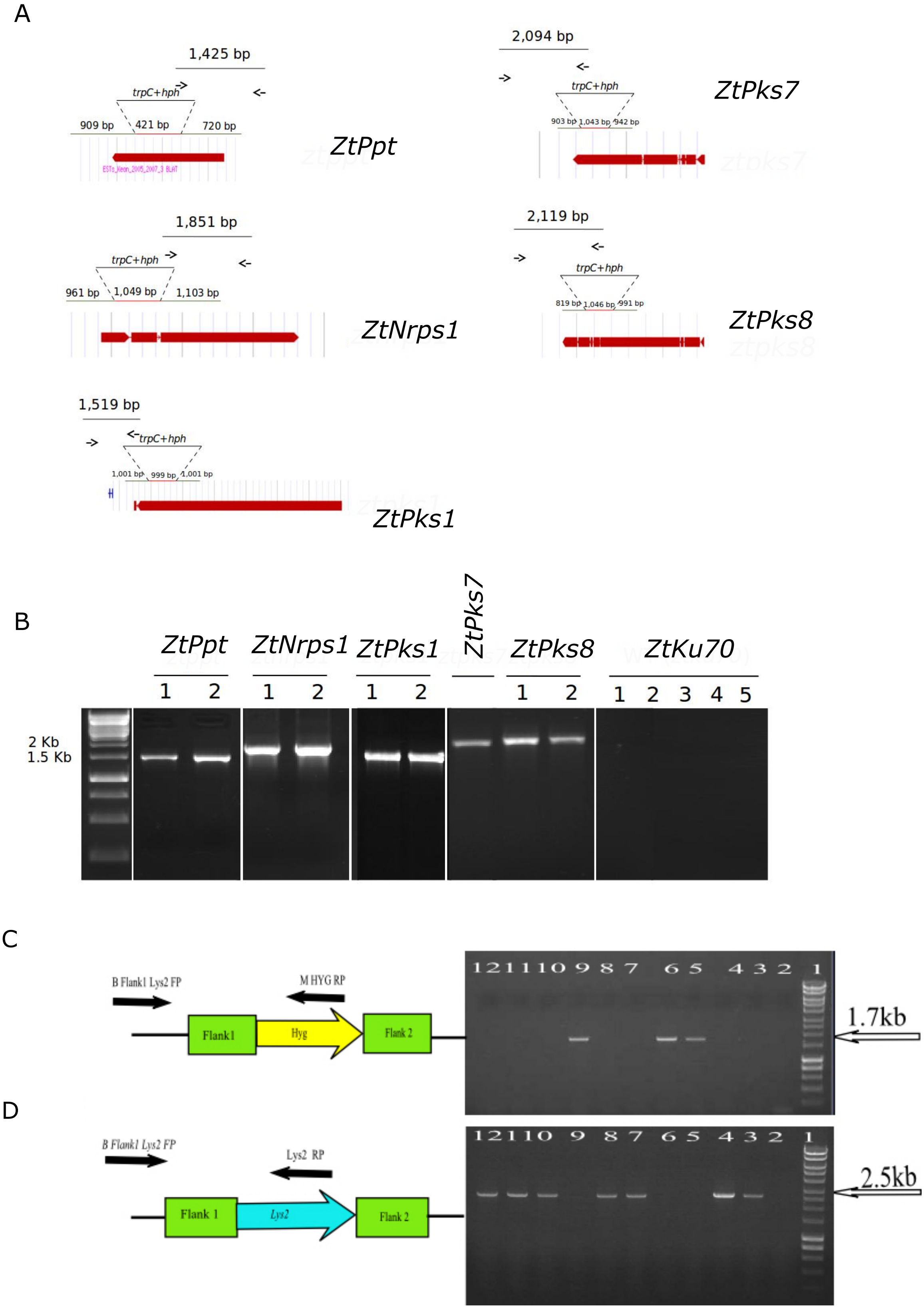

Supplementary Figure 1. Positions of flanking sequences for PPT and associated gene disruption, primers used for knockout confirmation and PCR agarose gel images confirming targeted integration. (A) Genes are represented by red arrows. Above each arrow, the lines represent the two flanking sequences with length in base pairs (bp) given above, the insertion position of the hph gene under the trpC promoter and positions of primers represented by arrows used to determine successful integration. **(B)** Composite PCR agarose gel showing amplification of regions targeted in mutant strains and WT genomic DNA. A single band of correct size was considered indicative of successful targeted gene disruption. **(C)** Diagram showing the primers used to confirm the *ZtAar (Lys2)* deletion. PCR amplification hygromycin B resistance gene, Lane 1 Hyperladder 1, lane 2 negative control D.W, lane 3 wild type IPO323. Lane 4 to 12; putative transformants. Lanes 5, 6 and 9 show expected bands of 1.7kb in Δ*ZtAar* mutants. **(D)** The pair of primers were used to confirm the presence of *Aar*. PCR amplification of *Aar* wild type locus, lane 5, 6 and 9 no band was seen indicating deletion of *Aar* gene.

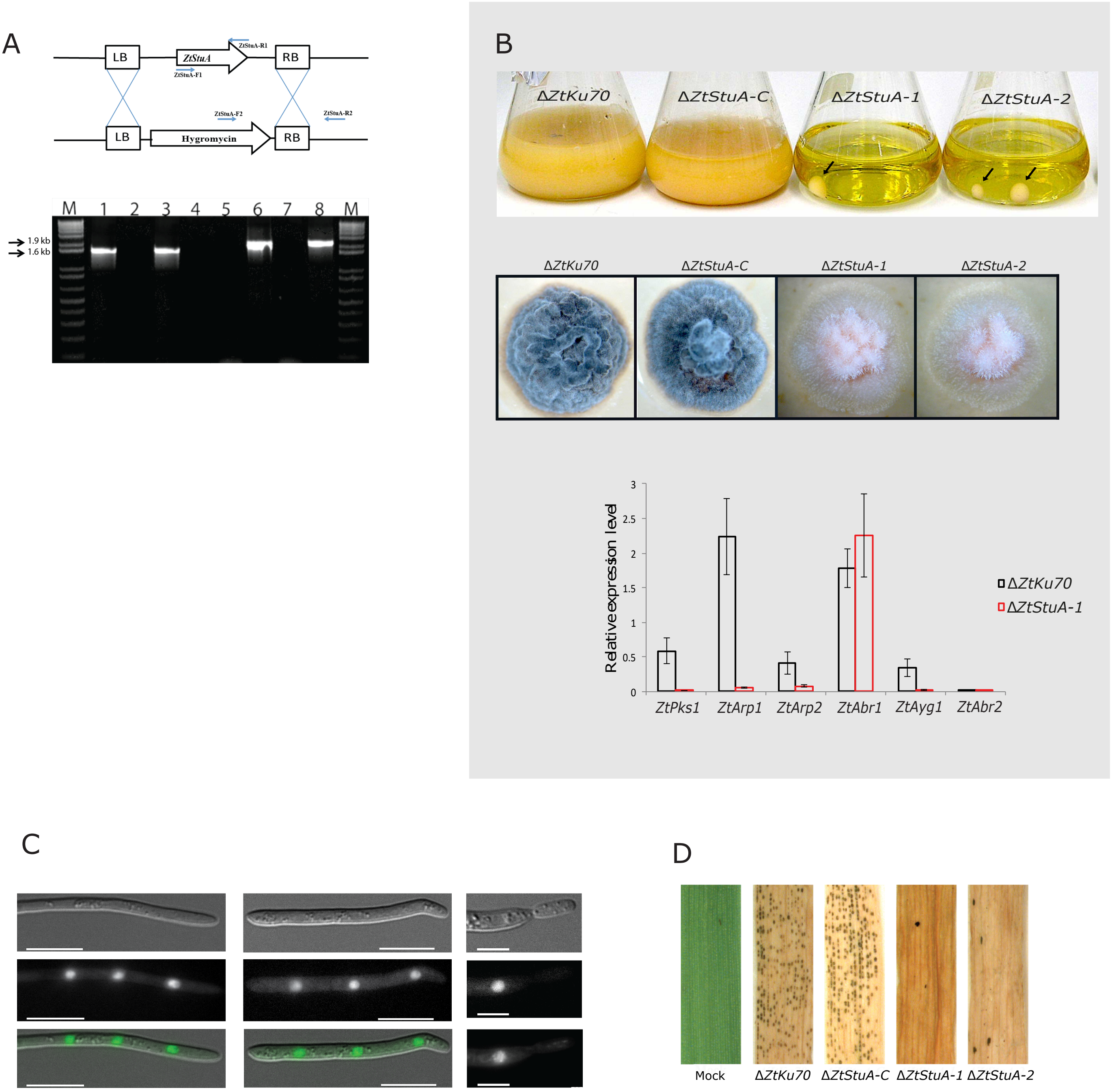

Supplementary Figure 2. Generation and functional characterisation of ΔZtStuA gene deletion strains (A) Validation of *ZtStuA* gene deletion strains. Diagram displaying the replacement by the *hygromycin phosphotransferase* (*hph*) resistance cassette through homologous recombination. The Δ*ZtKu70*, complemented strain as well as two independent mutant strains were used for PCR amplification using primers ZtStuA-F1, ZtStuA-R1, ZtStuA-F2 and ZtStuA-R2. Lanes 1 and 2 Z. tritici Δ*Ku70* (Δ*ZtKu70*). Lanes 3 and 4 Δ*ZtStuA-C*. Lanes 5 and 6 Δ*ZtStuA-1*. Lanes 7 and 8 Δ*ZtStuA-2*. Lane 1 shows the amplification of *ZtStuA* in Δ*ZtKu70* using primers ZtStuA-F1 and ZtStuA-R1 designed to amplify *ZtStuA* ORF whereas no amplicon of *ZtStuA* was observed in lane 2 with primers ZtStuA-F2 and ZtStuA-R2. Lane 3 shows the expected band of 1.6 kb in Δ*ZtStuA-C* amplified by using primers ZtStuA-F1 and ZtStuA-R1 whereas no amplicon of *ZtStuA* was observed in lane 4 with primers ZtStuA-F2 and ZtStuA-R2. Lane 5 indicates no amplicon of *ZtStuA* in Δ*ZtStuA-1* by using primers ZtStuA-F1 and ZtStuA-R1 while the expected band of 1.9 kb in lane 6 by using primers ZtStuA-F2 and ZtStuA-R2 was visualized. Lane 7 indicates no amplicon of *ZtStuA* ORF in Δ*ZtStuA-2* by using primers ZtStuA-F1 and ZtStuA-R1 while the expected band of 1.9 kb in lane 7 by using primers ZtStuA-F2 and ZtStuA-R2 was observed. **(B)** Effects on yeast-like cell production and biosynthesis of melanin. Upper panels- The Δ*ZtKu70* and Δ*ZtStuA-C* strains generated abundant yeast-like cells derived from blastic conidiogenesis in yeast glucose broth medium while Δ*ZtStuA-1* and 2 failed to sporulate and exclusively produced compact hyphal networks(marked with a black arrow). Middle panels- The Δ*ZtKu70* and Δ*ZtStuA-C* strains became melanizedwhereas the strains deleted for *ZtStuA* remained unmelanized. Lower panels- Comparative *in vitro* expression of putative genes involved in the melanization event in Δ*ZtKu70* versus the Δ*ZtStuA-1* strain. Error bars show standard deviation of the mean. **(C)** *ZtStuA* is localised to the nucleus. Left panels- The subcellular localization of the fluorescent protein ZtStuA::GFP was determined in yeast-like cells. Middle panels- localisation in hyphae; scale bars = 10 µm. Right panels- Yeast-like cells viewed under a fluorescent microscope with 4′,6-diamidino-2-phenylindole (DAPI) staining. Fluorescence co-localizes with the DAPI-stained nucleus; bars = 5 µm. (D) The effect of Zymoseptoria triici StuA (ZtStuA)
susceptible wheat cv. Taichung 29. Upper panels- From left to right, first leaves were inoculated with water (as a control) and the ΔZtKu70, ΔZtStuA-C, ΔZtStuA-1 and ΔZtStuA-2 strains. Final disease levels shown 20 days post inoculation (dpi).

Corresponding to Figure 2 *in vitro* filamentous growth assay (all strains that grew x 5 replicate plates)

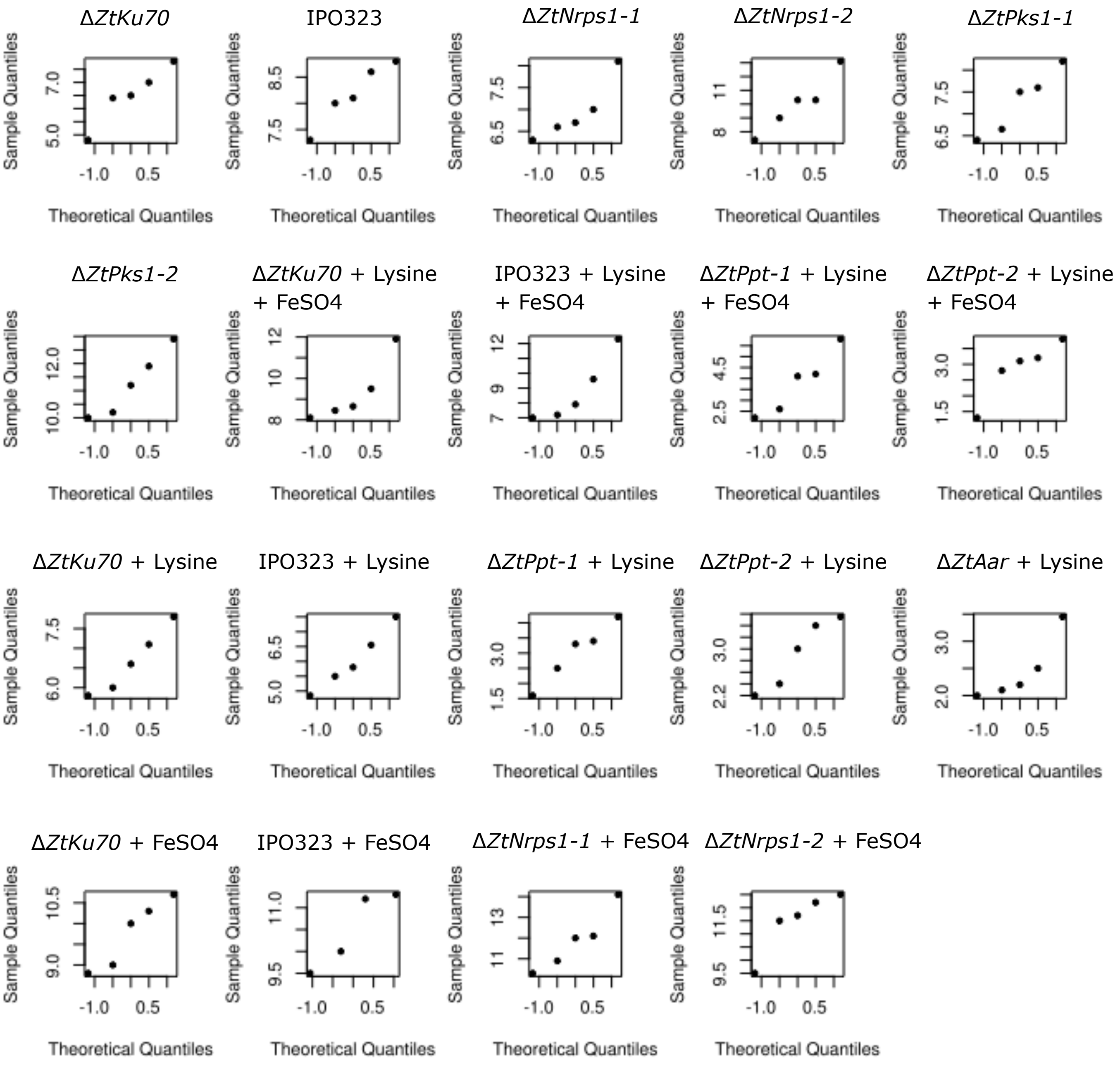

Supplementary Figure 3. Quantile plots of data from *in vitro* radial hyphal growth assay. Data are shown for all *in vitro* conditions tested using water agar for which more than 0 mm of radial growth was observed. Data were not transformed as they did not fail the Shapiro normality test at $\alpha = 0.05$.

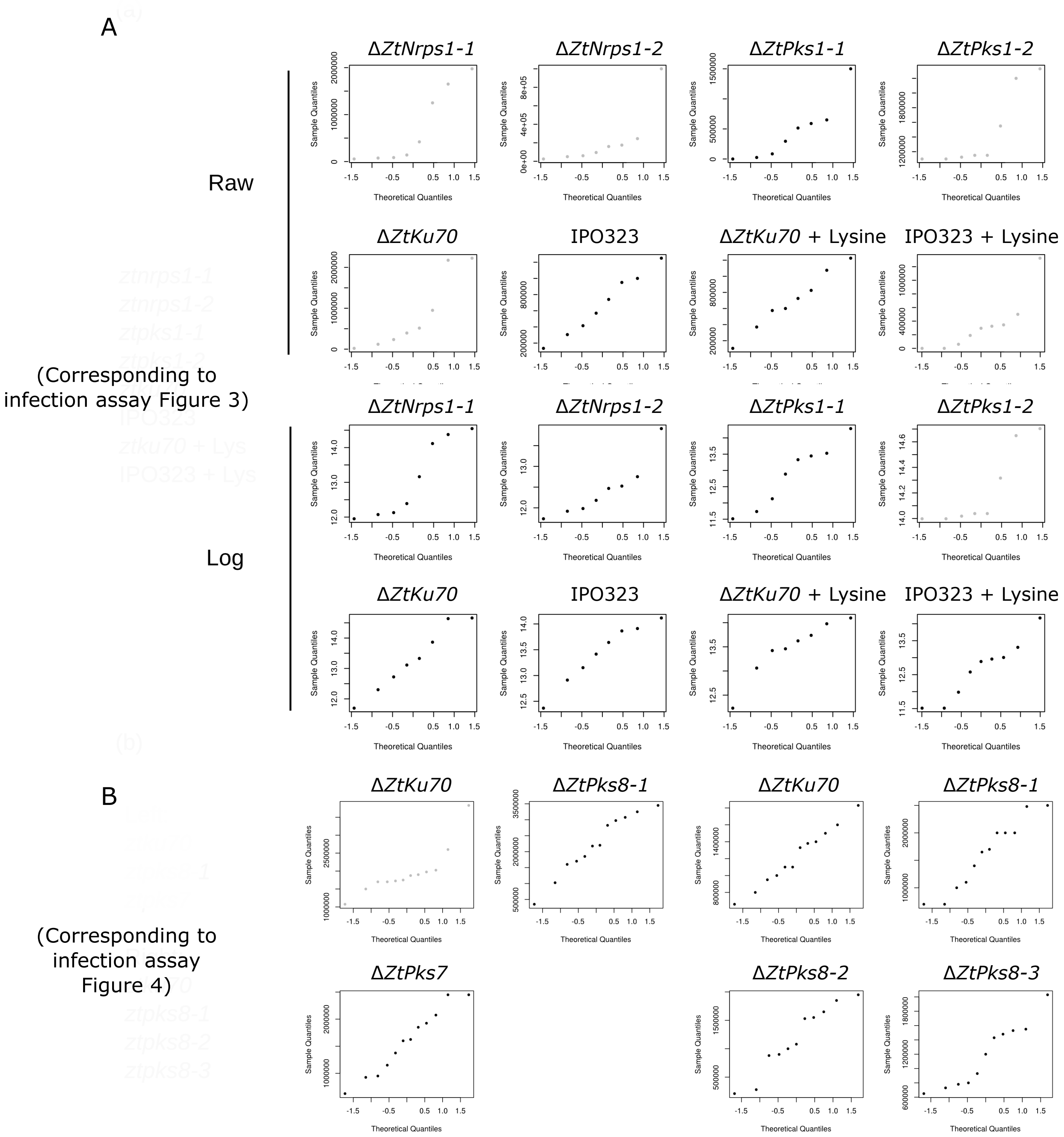

Supplementary Figure 4. Quantile plots of spore counts from samples of infected leaves. Data are shown for all strains that generated spores (several did not generate any spores, being completely avirulent, and were therefore omitted from statistical analysis) **(A)** Upper panel: Spore counts for all Ppt-associated strains and the two WTs after 22 dpi without lysine in the fungal inoculum. Grey points indicate a sample that failed the Shapiro normality test at $\alpha < 0.05$. Black points indicate data that were statistically likely to have come from a normally distributed population $\alpha > 0.05$. Lower panel: The same plots after data were log transformed. This reduced the number of samples that failed the Shapiro normality test at $\alpha < 0.05$. Grey points indicate a sample that failed the Shapiro normality test at $\alpha < 0.05$. Black points indicate data that were statistically likely to have come from a normally distributed population $\alpha > 0.05$. **(B)** Left: spore counts for the WT strain Δ*ZtKu70* and two of the PKS mutants, Δ*ZtPks7* and Δ*ZtPks8-1* after 22 dpi. Right: spore counts for the WT strain Δ*ZtKu70* and the three Δ*ZtPks8* strains tested. Data were not transformed.
Grey points indicate a sample that failed the Shapiro normality test at $\alpha < 0.05$. Black points indicate data that were statistically likely to have come from a normally distributed population $\alpha > 0.05$

Supplementary Table 1. ***Zymoseptoria tritici*** **has homologues of the PPT gene and associated genes characterised in** ***Cochliobolus sativus*****.**

Zymoseptoria tritici		*Cochliobolus sativus*			
**Gene name**	**GenBank accession**	**Gene name**	**GenBank accession**	**Amino acid identity (%)**	**E value**
ZtPpt	XP_003854473.1	*PPT1*	AER36018.1	41	3.00E-98
ZtNrps1	XP_003850202.1	*NPS6*	AER36015.1	33	0
ZtPks1	XP_003848644.1	*PKS1*	AER36016.1	63	0
ZtAar	XP_003855519.1	*AAR1*	AER36017.1	55	0
ZtPks7	XP_003850944.1	-	-	-	-
ZtPks8	XP_003847731.1	-	-	-	-

Supplementary Table 2. Homology of the *Zymoseptoria tritici* siderophore synthetase gene to siderophore synthetases from *Aspergillus fumigatus*.

Zymoseptoria tritici		*Aspergillus fumigatus*			
**Gene name**	**GenBank accession**	**Gene name**	**GenBank accession**	**Amino acid identity (%)**	**E value**
ZtNrps1	XP_003850202.1	*sidC*	XP_753088.1	25	6E-97
ZtNrps1	XP_003850202.1	*sidD*	XP_748662.1	36	0
ZtNrps1	XP_003850202.1	*sidF*	XP_748660.1	-	-
ZtNrps1	XP_003850202.1	*sidG*	XP_748685.1	-	-

Supplementary Table 3. ***Zymoseptoria tritici*** **has homologues of** ***Aspergillus fumigatus*** **genes encoded the DHN-melanin pathway.**

Gene	Enzyme activity	Size of deduced protein (amino acid)	Location	Identity	Reference gene
ZtPks1	Polyketide synthase	2176	Ch11	45%	Afalb1
ZtArp1	Scytalone dehydratase	222	Ch1	54%	Afarp1
ZtArp2	HN reductase	268	Ch11	50%	AfArp2
ZtAbr1	Multicopper oxidase	591	Ch2	42%	AfAbr1
ZtAyg1	Unknown	405	Ch3	53%	Afayg1
ZtAbr2	Laccase	591	Ch2	31%	Afabr2

Supplementary Table 4. Primers used in knockout construct generation and confirmation of homologous recombination.

Primer name	Target gene	Primer sequence	Use
4'-PPT-F1	*ZtPpt*	*(HindIII) AA AAG CTT* GTT CGA GCT GGT CTT GGA GCA GTC	KO construct generation
4'-PPT-R1	*ZtPpt*	*(XbaI) AA TCT AGA* ATT ACG GCG TTT GAC GAG AAC CAC	KO construct generation
4'-PPT-F2	*ZtPpt*	*(KpnI) AA GGT ACC* CGA TGT CAG TGC CCA CCA TGA CAT TCG TGT CAA AGT	KO construct generation
4'-PPT-R2	*ZtPpt*	*(SacI) AA GAG CTC* TCA CTT GTC AGC TCA GCG GTG CT	KO construct generation
PKS8-F1	*ZtPks8*	*(SacI) AA GAG CTC* GAC TCT CAA TGC CGG TAG CTG TGG	KO construct generation
PKS8-R1	*ZtPks8*	*(KpnI) AA GGT ACC* CAT GAG CTT CCC ATT GTT CAC GC	KO construct generation
PKS8-F2	*ZtPks8*	*(XbaI) AA TCT AGA* TGC GAC CTT GTC CAA CGA GTA GC	KO construct generation
PKS8-R2	*ZtPks8*	*(HindIII) AA AAG CTT* ATG GGA TAG GAC TGG CTG GGT CGT C	KO construct generation
PKS7-F1	*ZtPks7*	*(SacI) AA GAG CTC* TAC TAT ATA GCA AAA GAC AGC TTT TCC GTA ACA GC	KO construct generation
PKS7-R1	*ZtPks7*	*(KpnI) AA GGT ACC* TTT TCA ATT GAG CAG CAG GAC AAG G	KO construct generation
PKS7-F2	*ZtPks7*	*(XbaI) AA TCT AGA* GCT TCC CGA GCA AGC CGT GT	KO construct generation
PKS7-R2	*ZtPks7*	*(SalI) AA GTC GAC* TCG AGC AGG AGA TGG GTG CGA	KO construct generation
NRPS1-F1	*ZtNrps1*	*(SacI) AA GAG CTC* GCC TCG GGA TTT GGA AGA TGA CGT G	KO construct generation
NRPS1-R1	*ZtNrps1*	*(KpnI) AA GGT ACC* GGG CCA GGG CGT TTG CTT TG	KO construct generation
NRPS1-F2	*ZtNrps1*	*(XbaI) AA TCT AGA* TGC CCG ACG GCA AGT TGG AG	KO construct generation
NRPS1-R2	*ZtNrps1*	*(SalI) AA GTC GAC* TGC GTA AGG ACG GAC GCA GG	KO construct generation
PKS1-F1	*ZtPks1*	*(ApaI) AA GGG CCC* GTG AGA ACG TTC AAA CCG CC	KO construct generation
PKS1-R1	*ZtPks1*	*(KpnI) AA GGT ACC* GGC	KO construct generation

		CGA ATG CGA ACG TAT TC	
PKS1-F2	*ZtPks1*	*(XbaI) AA TCT AGA* AGG TCG ACT TGT CTT GGC TG	KO construct generation
PKS1-R2	*ZtPks1*	*(HindIII) AA AAG CTT* CAC AGG TGA TGT TGG ACC CA	KO construct generation
4'-PPT+Hyg-F	*ZtPpt*	CGG CGC AGC TAT TTA CCC GCA	Confirmation of targeted disruption
4'-PPT+Hyg-R	*ZtPpt*	TCG CTT TCA TCG TCG CCC CC	Confirmation of targeted disruption
NRPS1+Hyg-F	*ZtNrps1*	CTG CCC GCT GTT CTC CAG CC	Confirmation of targeted disruption
NRPS1+Hyg-R	*ZtNrps1*	TGC CAG AAG GTC GGA CCG GA	Confirmation of targeted disruption
PKS7+Hyg-F	*ZtPks7*	GCG GGT AAA TAG CTG CGC CGA	Confirmation of targeted disruption
PKS7+Hyg-R	*ZtPks7*	CGG TCT TCG CAG GAC GGC AA	Confirmation of targeted disruption
PKS8+Hyg-F	*ZtPks8*	ATG CAG CTC TCG GAG GGC GA	Confirmation of targeted disruption
PKS8+Hyg-R	*ZtPks8*	ACC GCT GGC GGA AAA GAC CG	Confirmation of targeted disruption
StuA-F1	*ZtStuA*	ATGTCTGCACAACCACAAC	Confirmation of targeted deletion
StuA-R1	*ZtStuA*	GGCTATGGGACGTGTGGAGA	Confirmation of targeted deletion
ZtStuA-F2	*ZtStuA*	GTGCTCACCGCCTGGACGACTAAAC	Confirmation of targeted deletion
ZtStuA-R2	*ZtStuA*	GATCTATGAGCGACTAGGAGG	Confirmation of targeted deletion
ZtStuA-F3	*ZtStuA*	GGTCTTAAUCCAATAGCACTCCCATTCGT	KO construct generation
ZtStuA-R3	*ZtStuA*	GGCATTAAUAGGCAAAGGCAATGATTGAG	KO construct generation
ZtStuA-F4	*ZtStuA*	GGACTTAAUTGTTGTCACGAAACGAAAGC	KO construct generation
ZtStuA-R4	*ZtStuA*	GGGTTTAAUTTCTCTGCCTCCTCAAAAGC	KO construct generation
MG-Sep-106	*ZtStuA*	CTGGTGGCAGGATATATTGTGGTGTAAACAATTAACGCCGAATTAATTCCTA	Complementation construct generation
MG-Sep-107	*ZtStuA*	CCCGCCAATATATCCTGTCAAA	Complementation construct generation
MG-Sep-113	*ZtStuA*	TATCAGTGTTTGACAGGATATATTGGCGGGTTGCAACTTGAAGCTGACCATA	Complementation construct generation
MG-Sep-116	*ZtStuA*	TAAACGCTCTTTTCTCTTAGGTTTACCCGCTAAGAGATTGGGAAGCAGCGAC	Complementation construct generation
SK-Sep-346	*ZtStuA/ZtGFP*	ATCACCCTCGGCATGGACGAGCTCTACAAGATGTCT	GFP fusion construct generation

		GCACAACCACAACCTCC	
SK-Sep-347	*ZtStuA/ZtGFP*	CCACAAGATCCTGTCCTC GTCCGTCGTCGCTTATCG CCTCATGCCGCCGGC	GFP fusion construct generation

Supplementary Table 5. Primers used in the expression analysis of melanin biosynthesis genes.

Name	Sequence (5-3)
Q-ZtStuA-F	TGCCTCAGAGCAACTTGAAC
Q-ZtStuA-R	GTATTGCGATGCCTGGTATG
Q-ZtPks1-F	TCTTGCCAGAACGTCATCAG
Q-ZtPks1-R	GCAGCGTTGTTTACCATGTG
Q-ZtArp1-F	GATTACCGCTCCTTCCTCAAC
Q-ZtArp1-R	CGATGAAGTGCTGGGTTTTC
Q-ZtArp2-F	ATGGCAAAGTTGCTCTCGTC
Q-ZtArp2-R	ACGGAATTGGCGTAGTTGAC
Q-ZtAbr1-F	TGAACGACACGCAGAATCTC
Q-ZtAbr1-R	TGTTGTGACCTTCGATCCAG
Q-ZtAyg1-F	AAGCCAAGGACCACGAATAC
Q-ZtAyg1-R	TCCGGTTTCAATGAGACTCC
Q-ZtAbr2-F	TCGAGAATGATCAGCAGGTG
Q-ZtAbr2-R	TCATGACCCAAAGCTTCCTC
